# Crystal structure of Na_4_Co_7−*x*_Al_0.67*x*_(As_1−*y*_P_*y*_O_4_)_6_ (*x* = 1.60; *y* = 0.116)

**DOI:** 10.1107/S205698901600400X

**Published:** 2016-03-15

**Authors:** Chokri Issaoui, Hammouda Chebbi, Abderrahmen Guesmi

**Affiliations:** a Université de Tunis El Manar, Faculté des Sciences, Laboratoire de Matériaux, Cristallochimie et Thermodynamique Appliquée, El Manar II, 2092 Tunis, Tunisia; b Université de Tunis, Institut Préparatoire aux Etudes d’Ingénieurs de Tunis, Rue Jawaher Lel Nehru, 1089 Montfleury, Tunis, Tunisia; c Al-Baha University, Faculty of Sciences and Arts in Al Mukhwah, Al Mukhwah, Al Baha Region, Kingdom of Saudi Arabia

**Keywords:** crystal structure, Na_4_Co_5.40_Al_1.07_(As_0.883_P_0.116_O_4_)_6_, bond-valence sum, charge distribution

## Abstract

The title compound, tetra­sodium hepta­(cobalt/aluminium) hexa­(arsenate/phosphate), Na_4_Co_5.40_Al_1.07_(As_0.883_P_0.116_O_4_)_6_, was prepared by a solid-state reaction. It is a new member of the family of isostructural compounds with the general formula *A*_4_ *M*_7_(*X*O_4_)_6_ (*A*: Na, K; *M*: Ni, Co; *X*: P, As) that is most similar to Na_4_Co_5.63_Al_0.91_(AsO_4_)_6_. The Co^2+^ ions in the title compound are substituted by Al^3+^ in a fully occupied octa­hedral site (site symmetry 2/*m*) and a partially occupied tetra­hedral site (site symmetry 2). A third octa­hedral site is fully occupied by Co^2+^ ions only. With regard to the P and As atoms, one site (site symmetry *m*) is simultaneously occupied by As and P, whereas in the second site there is only arsenic. The alkali cations are, as in the isostructural compounds, distributed over half-occupied crystallographic sites, with a positional disorder of one of them. The proposed structural model is based both on a careful investigation of the crystal data, as well as validation by means of bond-valence-sum (BVS) and charge-distribution (CHARDI) calculations. The correlation between the X-ray refinement and the validation results is discussed.

## Abstract 2

Tetra­sodium hepta­(cobalt/aluminium) hexa­(arsenate/phosphate) is a new member of the isostructural family of compounds with the general formula *A*_4_ *M*_7_(*X*O_4_)_6_ (*A*: Na, K; *M*: Ni, Co; *X*: P, As). The proposed structural model is based both on a careful investigation of the crystal data, as well as validation tools by means of bond-valence-sum (BVS) and charge-distribution (CHARDI) calculations.

## Chemical context   

Metal-substituted aluminophosphates and aluminoarsenates form an important group of materials with many inter­esting properties such as mol­ecular sieves, catalysts, *etc*. Li *et al.* (2012 ▸) reported the progress in heteroatom-containing alumino­phosphate mol­ecular sieves. With regard to their As homologues, one can cite AlAsO_4_-5 and AlAsO_4_-6, two aluminoarsenates with occluded ethyl­enedi­amine (Chen *et al.* 1990 ▸). The analogous cobalt compounds, such as ammonium-templated cobalt aluminophosphates with zeolite-like structures (Bontchev & Sevov, 1999 ▸), possess similar structural properties.

**Figure 1:**
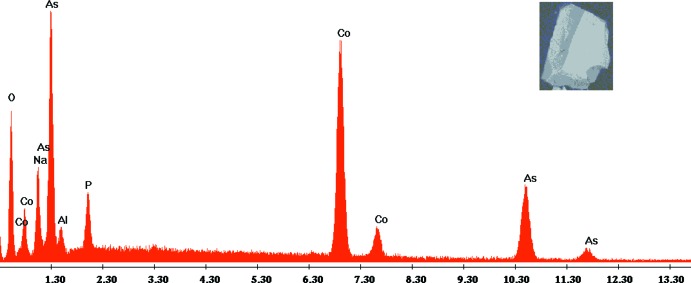
The EDX spectrum of the title compound. The inset shows the morphology of one crystal.

The title compound, Na_4_Co_7−*x*_Al_0.67*x*_(As_1−*y*_P_*y*_O_4_)_6_ (*x* = 1.60; *y* = 0.116), was obtained during the exploration of the Na–Co–P–As–O system by solid-state reaction; as for many aluminophosphates, aluminum was incorporated from the reaction container. The chemical composition and crystal structure were determined by energy-dispersive X-ray spectroscopy (EDX) analysis (Fig. 1 ▸) and single-crystal X-ray diffraction; the proposed structural model is supported by validation tools by means of bond-valence-sum (BVS) calculations and charge-distribution (CHARDI) analysis (Brown, 2002 ▸; Adams, 2003 ▸, Nespolo, 2015 ▸, 2016 ▸; Eon & Nespolo, 2015 ▸). The correlation between the experimental and the validation results is discussed.

## Structural commentary   

The title compound is a new member of the isostructural compounds family with the general formula *A*_4_ *M*_7_(*X*O_4_)_6_ (*A*: Na, K; *M*: Ni, Co; *X*: P, As) (Moring & Kostiner, 1986 ▸; Kobashi *et al.*, 1998 ▸; Ben Smail *et al.*, 1999 ▸; Marzouki *et al.*, 2010 ▸, 2013 ▸).

**Figure 2:**
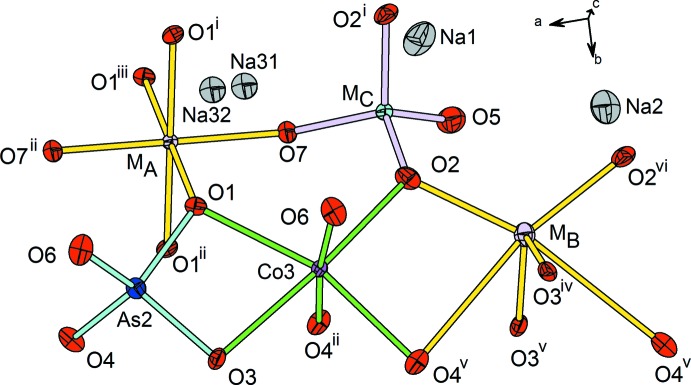
The asymmetric unit of (I), showing the atom-labelling scheme. The full coordination polyhedra are shown, including the corresponding symmetry-related O atoms. Displacement ellipsoids are drawn at the 50% probability level. [*M*_A_ = Co_0.189_Al_0.811_; *M*_B_ = Co_0.605_Al_0.135_□_0.260_; *M*_C_ = As_0.65_P_0.35_. Symmetry codes: (i) *x*, −*y*, *z*; (ii) −*x*, *y*, −*z*; (iii) −*x*, −*y*, −*z*; (iv) −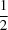 − *x*, 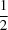 − *y*, *z*; (v) −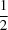 − *x*, 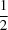 − *y*, −*z*; (vi) −1 − *x*, *y*, −*z*.]

The asymmetric unit of the title compound (I) (Fig. 2 ▸) contains seven metallic sites of which four are occupied by Na^+^ cations (occupancies ranging from 0.23 to 0.50) with eight cations per unit cell, two others (denoted *M*_A_ and *M*_B_) are simultaneously shared by Co^2+^ and Al^3+^ ions, and one is fully occupied by Co^2+^ ions: the same distribution is observed in the homologous arsenate Na_4_Co_7−*x*_Al_0.67*x*_(AsO_4_)_6_ (*x* = 1.37) (II) (Marzouki *et al.*, 2010 ▸).

## Validation of the structural model using BVS and CHARDI   

Two validation tools, BVS and CHARDI, are used to support and analyse the proposed structural model. Briefly, for a properly refined structure, the valences *V* according to the BVS model and charges *Q* from the CHARDI analysis should agree with the oxidation states of the atoms (Brown, 2002 ▸; Adams, 2003 ▸, Nespolo, 2015 ▸, 2016 ▸; Eon & Nespolo, 2015 ▸).

The *M*_A_ site, with an octa­hedral environment by oxygen atoms, is fully occupied by the two cations with overall occupancy Co_0.189_Al_0.811_. This distribution scheme is confirmed by the validation tools, with a better convergence with the CHARDI model (Table 1 ▸). If compared to the homologous site in (II) with overall occupancy Co_0.286_Al_0.714_ (Marzouki *et al.*, 2010 ▸), the average arithmetic distance in (I) (1.91 Å) is smaller than in (II) (1.96 Å) due to the higher fraction of the small cation (Al^3+^) in (I).

For the *M*_B_ site with a tetra­hedral coordination, the Co^2+^/Al^3+^ distribution is based on the same observations as in (II), mainly if it is refined as partially occupied by just Co^2+^, the charge neutrality is not achieved, and then a fraction of Al^3+^ was introduced in the *M*_B_ site yielding an overall occupancy distribution of Co_0.605_Al_0.135_□_0.260_, with □ expressing the vacancy. The validation results for this particular distribution are: *V*(*M*_B_) = 1.31 and *Q*(*M*_B_) = 1.58, the theoretical value is 1.61 (Table 1 ▸). Finally, with regard to P and As atoms, the P/As substitutional disorder is observed in one of the two sites (*M*_C_): P/As = 0.35/0.65; *V* = 5.21 and *Q* = 5.00.

The final result corresponds to the formula Na_4_Co_5.40_Al_1.07_(As_0.883_P_0.116_O_4_)_6_. It is the first case in its homologous family which contains such a number of elements. The similarity to (II) (Marzouki *et al.*, 2010 ▸) is clear, the cell parameters of (I) are smaller than those of (II) as it contains more small elements than (II). The CHARDI method is extended, as for (II), to analyse the coordination polyhedra by means of the Effective Coordination Numbers (ECoN): the polyhedron distortion is more pronounced if the ECoN deviates more from the classical coordination number (CN).

**Figure 3:**
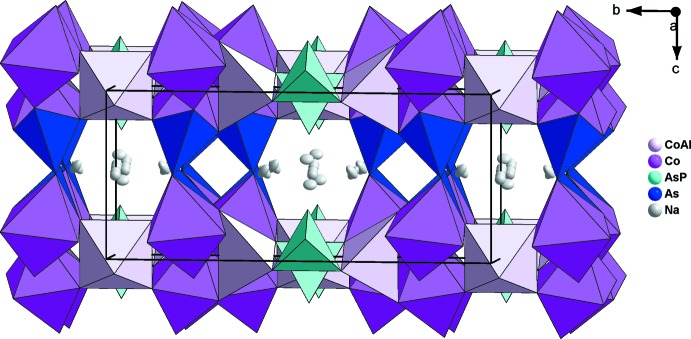
The structure of the title compound viewed appoximately along [100], showing the tunnels and the Na^+^ cations.

The framework of the title compound is of an open character (Fig. 3 ▸). Its aptitude for sodium conduction through the tunnels appears to be possible, as shown in experimental and theoretical studies for the similar compound (II) (Marzouki *et al.*, 2013 ▸). These studies will be the subject of future works.

## Synthesis and crystallization   

A mixture of sodium nitrate, cobalt nitrate hexa­hydrate, NH_4_H_2_ *X*O_4_ (*X*: P, As) in the molar ratio Na:Co:P:As = 2:1:0.5:1 was dissolved in deionized water and then heated at 373 K to dehydration. After grinding, it was placed in a porcelain boat and first heated at 673 K in air for 24 h and then heated gradually to 1123 K for 1 d. Some pink parallelepiped-shaped crystals were isolated from the sample. A qualitative EDX analysis confirmed the presence of Na, Co, Al, As and O (Fig. 1 ▸), with the aluminium diffusing from the reaction container.

## Refinement   

Crystal data, data collection and structure refinement details are summarized in Table 2 ▸. The Co and Al atoms occupying the *M*_A_ and *M*_B_ sites, as well as the P and As atoms occupying the *M*_C_ site, were constrained using the EXYZ and EADP instructions of *SHELXL97* (Sheldrick, 2008 ▸). Three linear free variable restraints (SUMP) were required to restrain the sum of their occupation factors. The Na1 and Na2 cations are at half-occupancy sites and the two others (Na31 and Na32) with isotropic refinement have a total occupancy of 0.50 because, when refined freely, their occupations converged to these values.

## Supplementary Material

Crystal structure: contains datablock(s) I. DOI: 10.1107/S205698901600400X/br2258sup1.cif

Structure factors: contains datablock(s) I. DOI: 10.1107/S205698901600400X/br2258Isup2.hkl

CCDC reference: 1462882

Additional supporting information:  crystallographic information; 3D view; checkCIF report

## Figures and Tables

**Table 1:** BVS and CHARDI analysis of cation polyhedra in the title compound (the structure described as being built of cation-centred polyhedra)

Cation	*q*(*i*)·sof*i*	*Vi*	*Qi*	CN*i*	ECoN*i*
*M*_A_	2.81	2.97	2.91	6	5.92
*M*_B_	1.61	1.31	1.58	4	3.95
Co3	2.00	2.05	1.99	6	5.88
*M*_C_	5.00	5.21	5.00	4	3.97
As2	5.00	5	5.09	4	3.98
Na1	0.50	0.51	0.49	5	4.53
Na2	0.50	0.52	0.49	7	6.18
Na31	0.23	0.23	0.23	7	6.06
Na32	0.27	0.28	0.27	6	5.31

Notes: *M*_A_ = Co_0.189_Al_0.811_; *M*_B_ = Co_0.605_Al_0.135_□_0.260_; *M*_c_ = As_0.65_P_0.35_; *q* is the formal oxidation number; sof is the site-occupation factor; MAPD = 1% [the mean absolute percentage deviation MAPD measures the agreement between *q* and *Q*; for more information, see Nespolo (2016 ▸)].

**Table 2:** Experimental details

Crystal data	
Chemical formula	Na_4_Co_5.40_Al_1.07_(As_0.883_P_0.116_O_4_)_6_
*M*_r_	1242.08
Crystal system, space group	Monoclinic, *C*2/*m*
Temperature (K)	293
*a*, *b*, *c* (Å)	10.5797 (2), 14.5528 (3), 6.6441 (3)
β (°)	105.608 (9)
*V* (Å^3^)	985.23 (7)
*Z*	2
Radiation type	Mo *K*α
μ (mm^−1^)	13.60
Crystal size (mm)	0.30 × 0.20 × 0.20

Data collection	
Diffractometer	Enraf–Nonius CAD-4
Absorption correction	ψ scan (North *et al.*, 1968 ▸)
*T*_min_, *T*_max_	0.055, 0.140
No. of measured, independent and observed [*I* > 2σ(*I*)] reflections	2409, 1124, 894
*R*_int_	0.027
(sin θ/λ)_max_ (Å^−1^)	0.638

Refinement	
*R*[*F* ^2^ > 2σ(*F* ^2^)], *wR*(*F* ^2^), *S*	0.030, 0.083, 1.07
No. of reflections	1124
No. of parameters	117
No. of restraints	2
Δρ_max_, Δρ_min_ (e Å^−3^)	0.81, −0.85

Computer programs: *CAD-4 EXPRESS* (Duisenberg, 1992 ▸; Macíček & Yordanov, 1992 ▸), *XCAD4* (Harms & Wocadlo, 1995 ▸), *SHELXS97* and *SHELXL97* (Sheldrick, 2008 ▸), *DIAMOND* (Brandenburg, 2006 ▸), *WinGX* (Farrugia, 2012 ▸) and *publCIF* (Westrip, 2010 ▸).

## supplementary crystallographic information

### Crystal data

**Table:**

Na_4_Co_5.40_Al_1.07_(As_0.883_P_0.116_O_4_)_6_	*F*(000) = 1162
*M**_r_* = 1242.08	*D*_x_ = 4.187 Mg m^−^^3^
Monoclinic, *C*2/*m*	Mo *K*α radiation, λ = 0.71073 Å
*a* = 10.5797 (2) Å	Cell parameters from 25 reflections
*b* = 14.5528 (3) Å	θ = 12.0–14.8°
*c* = 6.6441 (3) Å	µ = 13.60 mm^−^^1^
β = 105.608 (9)°	*T* = 293 K
*V* = 985.23 (7) Å^3^	Parallelepiped, pink
*Z* = 2	0.30 × 0.20 × 0.20 mm

### Data collection

**Table:**

Enraf–Nonius CAD-4 diffractometer	*R*_int_ = 0.027
ω/2θ scans	θ_max_ = 27.0°, θ_min_ = 2.4°
Absorption correction: ψ scan (North *et al.*, 1968)	*h* = −13→13
*T*_min_ = 0.055, *T*_max_ = 0.140	*k* = −1→18
2409 measured reflections	*l* = −8→8
1124 independent reflections	2 standard reflections every 120 reflections
894 reflections with *I* > 2σ(*I*)	intensity decay: 1%

### Refinement

**Table:**

Refinement on *F*^2^	117 parameters
Least-squares matrix: full	2 restraints
*R*[*F*^2^ > 2σ(*F*^2^)] = 0.030	*w* = 1/[σ^2^(*F*_o_^2^) + (0.0401*P*)^2^ + 10.5538*P*] where *P* = (*F*_o_^2^ + 2*F*_c_^2^)/3
*wR*(*F*^2^) = 0.083	(Δ/σ)_max_ < 0.001
*S* = 1.07	Δρ_max_ = 0.81 e Å^−^^3^
1124 reflections	Δρ_min_ = −0.85 e Å^−^^3^

### Special details

**Table:**

Geometry. All e.s.d.'s (except the e.s.d. in the dihedral angle between two l.s. planes) are estimated using the full covariance matrix. The cell e.s.d.'s are taken into account individually in the estimation of e.s.d.'s in distances, angles and torsion angles; correlations between e.s.d.'s in cell parameters are only used when they are defined by crystal symmetry. An approximate (isotropic) treatment of cell e.s.d.'s is used for estimating e.s.d.'s involving l.s. planes.

### Fractional atomic coordinates and isotropic or equivalent isotropic displacement parameters (Å^2^)

**Table:**

	*x*	*y*	*z*	*U*_iso_*/*U*_eq_	Occ. (<1)
Co1	0.0000	0.0000	0.0000	0.0064 (9)	0.189 (13)
Al1	0.0000	0.0000	0.0000	0.0064 (9)	0.811 (13)
Co2	−0.5000	0.16324 (13)	0.0000	0.0118 (6)	0.605 (9)
Al2	−0.5000	0.16324 (13)	0.0000	0.0118 (6)	0.135 (9)
Co3	−0.18046 (7)	0.18027 (5)	0.17925 (10)	0.0062 (2)	
As1	−0.32397 (10)	0.0000	−0.06479 (16)	0.0091 (4)	0.649 (7)
P1	−0.32397 (10)	0.0000	−0.06479 (16)	0.0091 (4)	0.351 (7)
As2	0.09963 (5)	0.17931 (4)	0.29004 (8)	0.00940 (18)	
Na1	−0.4220 (5)	−0.1148 (4)	−0.5048 (8)	0.0258 (12)	0.5
Na2	−0.6741 (7)	0.0000	−0.4195 (11)	0.0217 (16)	0.5
Na31	−0.084 (3)	0.0000	0.469 (3)	0.017 (2)*	0.229 (19)
Na32	−0.036 (2)	0.0000	0.487 (2)	0.017 (2)*	0.271 (19)
O1	−0.0101 (4)	0.0937 (3)	0.2026 (6)	0.0090 (8)	
O2	−0.3346 (4)	0.0895 (3)	0.0802 (6)	0.0127 (8)	
O3	−0.0063 (4)	0.2670 (3)	0.2696 (6)	0.0100 (8)	
O4	0.1921 (4)	0.2070 (3)	0.1327 (6)	0.0111 (8)	
O5	−0.4356 (6)	0.0000	−0.2789 (10)	0.0202 (14)	
O6	0.1900 (4)	0.1511 (3)	0.5228 (6)	0.0134 (9)	
O7	−0.1813 (6)	0.0000	−0.1116 (10)	0.0141 (13)	

### Atomic displacement parameters (Å^2^)

**Table:**

	*U*^11^	*U*^22^	*U*^33^	*U*^12^	*U*^13^	*U*^23^
Co1	0.0061 (14)	0.0053 (15)	0.0075 (14)	0.000	0.0013 (9)	0.000
Al1	0.0061 (14)	0.0053 (15)	0.0075 (14)	0.000	0.0013 (9)	0.000
Co2	0.0103 (8)	0.0146 (10)	0.0102 (9)	0.000	0.0021 (6)	0.000
Al2	0.0103 (8)	0.0146 (10)	0.0102 (9)	0.000	0.0021 (6)	0.000
Co3	0.0065 (3)	0.0070 (4)	0.0048 (3)	0.0004 (3)	0.0007 (3)	0.0005 (3)
As1	0.0070 (5)	0.0067 (6)	0.0131 (6)	0.000	0.0019 (4)	0.000
P1	0.0070 (5)	0.0067 (6)	0.0131 (6)	0.000	0.0019 (4)	0.000
As2	0.0090 (3)	0.0115 (3)	0.0070 (3)	−0.0007 (2)	0.0010 (2)	0.0008 (2)
Na1	0.026 (3)	0.020 (3)	0.027 (3)	0.005 (2)	0.001 (2)	−0.011 (2)
Na2	0.022 (4)	0.029 (5)	0.018 (4)	0.000	0.012 (3)	0.000
O1	0.0112 (17)	0.0083 (19)	0.0072 (18)	−0.0014 (15)	0.0018 (14)	−0.0006 (16)
O2	0.0149 (19)	0.011 (2)	0.0122 (19)	−0.0039 (16)	0.0043 (15)	−0.0052 (17)
O3	0.0076 (18)	0.010 (2)	0.0112 (19)	0.0023 (16)	0.0002 (14)	−0.0004 (16)
O4	0.0150 (19)	0.016 (2)	0.0041 (18)	−0.0056 (17)	0.0050 (15)	−0.0040 (16)
O5	0.019 (3)	0.016 (4)	0.022 (3)	0.000	0.000 (3)	0.000
O6	0.0145 (19)	0.023 (2)	0.0022 (17)	0.0030 (18)	0.0009 (15)	−0.0017 (16)
O7	0.009 (3)	0.011 (3)	0.023 (3)	0.000	0.006 (2)	0.000

### Geometric parameters (Å, º)

**Table:**

Co1—O7^i^	1.861 (6)	Na2—Na1^ix^	2.086 (8)
Co1—O7	1.861 (6)	Na2—Na1^xi^	2.086 (8)
Co1—O1	1.939 (4)	Na2—O5	2.443 (10)
Co1—O1^i^	1.939 (4)	Na2—Na31^xii^	2.49 (3)
Co1—O1^ii^	1.939 (4)	Na2—O5^ix^	2.572 (10)
Co1—O1^iii^	1.939 (4)	Na2—O2^iv^	2.584 (7)
Co2—O2^iv^	1.999 (4)	Na2—O2^xii^	2.584 (7)
Co2—O2	1.999 (4)	Na2—O6^xiii^	2.598 (6)
Co2—O3^v^	2.075 (4)	Na2—O6^xiv^	2.598 (6)
Co2—O3^vi^	2.075 (4)	Na2—Na32^xii^	2.98 (2)
Co3—O6^vii^	2.054 (4)	Na31—Na32^xv^	1.23 (5)
Co3—O2	2.064 (4)	Na31—Na31^xv^	1.72 (6)
Co3—O4^iii^	2.080 (4)	Na31—O6^xv^	2.473 (15)
Co3—O4^v^	2.092 (4)	Na31—O6^vii^	2.473 (15)
Co3—O1	2.171 (4)	Na31—Na2^xii^	2.49 (3)
Co3—O3	2.181 (4)	Na31—O1	2.524 (17)
As1—O5	1.586 (6)	Na31—O1^ii^	2.524 (17)
As1—O7	1.621 (6)	Na31—O1^vii^	2.536 (17)
As1—O2^ii^	1.642 (4)	Na31—O1^xv^	2.536 (17)
As1—O2	1.642 (4)	Na32—Na32^xv^	0.73 (4)
As2—O6	1.637 (4)	Na32—Na31^xv^	1.23 (5)
As2—O4	1.662 (4)	Na32—O1^vii^	2.408 (13)
As2—O3	1.680 (4)	Na32—O1^xv^	2.408 (13)
As2—O1	1.695 (4)	Na32—O1	2.407 (13)
Na1—O5	2.276 (7)	Na32—O1^ii^	2.407 (13)
Na1—O3^viii^	2.298 (7)	Na32—O6^xv^	2.727 (14)
Na1—O5^ix^	2.441 (7)	Na32—O6^vii^	2.727 (14)
Na1—O6^i^	2.545 (7)	Na32—Na2^xii^	2.98 (2)
Na1—O3^x^	2.572 (8)		
			
O7^i^—Co1—O7	180.0	O2^iv^—Co2—O3^vi^	105.15 (15)
O7^i^—Co1—O1	88.09 (17)	O2—Co2—O3^vi^	105.29 (15)
O7—Co1—O1	91.91 (17)	O3^v^—Co2—O3^vi^	121.5 (2)
O7^i^—Co1—O1^i^	91.91 (17)	O6^vii^—Co3—O2	86.29 (16)
O7—Co1—O1^i^	88.09 (17)	O6^vii^—Co3—O4^iii^	173.90 (16)
O1—Co1—O1^i^	180.0	O2—Co3—O4^iii^	88.41 (16)
O7^i^—Co1—O1^ii^	88.09 (17)	O6^vii^—Co3—O4^v^	96.19 (16)
O7—Co1—O1^ii^	91.91 (17)	O2—Co3—O4^v^	91.84 (17)
O1—Co1—O1^ii^	89.4 (2)	O4^iii^—Co3—O4^v^	80.95 (17)
O1^i^—Co1—O1^ii^	90.6 (2)	O6^vii^—Co3—O1	93.80 (16)
O7^i^—Co1—O1^iii^	91.91 (17)	O2—Co3—O1	102.77 (16)
O7—Co1—O1^iii^	88.09 (17)	O4^iii^—Co3—O1	90.31 (15)
O1—Co1—O1^iii^	90.6 (2)	O4^v^—Co3—O1	162.79 (16)
O1^i^—Co1—O1^iii^	89.4 (2)	O6^vii^—Co3—O3	96.40 (16)
O1^ii^—Co1—O1^iii^	180.0 (3)	O2—Co3—O3	174.29 (16)
O2^iv^—Co2—O2	115.1 (3)	O4^iii^—Co3—O3	89.15 (16)
O2^iv^—Co2—O3^v^	105.29 (15)	O4^v^—Co3—O3	92.87 (16)
O2—Co2—O3^v^	105.15 (15)	O1—Co3—O3	72.08 (15)

Symmetry codes: (i) −*x*, −*y*, −*z*; (ii) *x*, −*y*, *z*; (iii) −*x*, *y*, −*z*; (iv) −*x*−1, *y*, −*z*; (v) *x*−1/2, −*y*+1/2, *z*; (vi) −*x*−1/2, −*y*+1/2, −*z*; (vii) −*x*, *y*, −*z*+1; (viii) *x*−1/2, *y*−1/2, *z*−1; (ix) −*x*−1, −*y*, −*z*−1; (x) −*x*−1/2, *y*−1/2, −*z*; (xi) −*x*−1, *y*, −*z*−1; (xii) −*x*−1, −*y*, −*z*; (xiii) *x*−1, −*y*, *z*−1; (xiv) *x*−1, *y*, *z*−1; (xv) −*x*, −*y*, −*z*+1.
